# Interview with Prof. Dr. Jean-Marc Burgunder - 8^th^ European Congress on Neurorehabilitation in conjunction with the 20^th^ Congress of the Society for the Study of Neuroprotection and Neuroplasticity

**DOI:** 10.25122/jml-2026-1002

**Published:** 2026-01

**Authors:** Stefana-Andrada Dobran, Alexandra Gherman

**Affiliations:** 1 RoNeuro Institute for Neurological Research and Diagnostic, Cluj-Napoca, Romania; 2 Sociology Department, Babes-Bolyai University, Cluj-Napoca, Romania

**Interviewee: Professor Dr. Jean-Marc Burgunder**

**Interviewer: Ms. Stefana-Andrada Dobran**

Professor Dr. med. Jean-Marc Burgunder is a senior consultant neurologist at Privatklinik Siloah and a specialist in neurology. With extensive international academic experience, he has held professorial and visiting positions at leading universities in Europe and Asia, including the University of Bern, the National University of Singapore, and Sun Yat-sen University. His clinical and research expertise spans neurology and neurogenetics, and his work has been recognized through multiple international awards in stroke and neurological research.

**S.D.: Hello, Professor Jean-Marc Burgunder, and welcome to the 8^th^ European Congress of Neurorehabilitation (ECNR) in conjunction with the 20^th^ Congress of the Society for the Study of Neuroprotection and Neuroplasticity. The ECNR brings together scientific and clinical communities. What do you believe is the unique role it plays in bridging the gap between research and daily patient care in neurorehabilitation?**

J.M.B.: Well, daily clinical care in neurology in general—and this is true for neurorehabilitation as well—has a very important role in bridging knowledge from basic science and translational science and bringing it into the clinic. So, there is a crucial interaction between both sides.

On one hand, you need to have clinical questions being raised, and once you have those questions, you can train them and conduct translational research. The other direction is also very important: that people in translational research explain their results to clinicians, so that together they can see whether those findings can be applied to clinical research, and even more directly, to clinical care in rehabilitation.


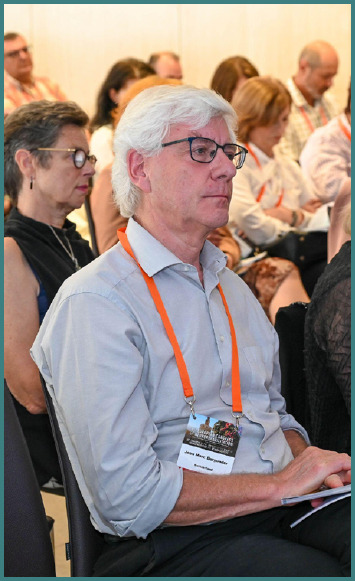


**S.D.: Considering your specialty, what future developments do you envision for the complex multidisciplinary field of neurorehabilitation?**

J.M.B.: That is a very interesting question. There is a lot happening now in neuroscience—particularly in terms of deep learning and new methods for understanding large data sets. I think neurology is a field where you can get the most out of this complexity of data. There are clinical data, imaging data across several modalities, and of course molecular and genetic data as well. To bring all of this together using these new methods is something very, very interesting.

Other important developments are, of course, technical—having better tools to aid in rehabilitation, especially in the context of decreasing availability or difficulty in finding qualified personnel to perform rehabilitation, or in places where staff is simply unavailable. The use of digital methods, including internet-based tools, will be very fruitful, and I hope that some areas in neurology will be particularly open to adopting them for this purpose.

**S.D.: Is there a specific trend or technology that you are most interested in?**

J.M.B.: There are so many possibilities that it’s difficult to choose just one, but I'm thinking about speech rehabilitation. In this area, you could assess speech in people with neurological disorders in general, and then use a system—like a digital agent—that responds directly and provides cues to the patient for specific exercises, such as breathing and speaking. That’s one example, but there could certainly be several others.

**S.D.: In your perspective, what is the most challenging future development in neurorehabilitation and how can the European Federation of Neurorehabilitation Societies (EFNR) help in this endeavor?**

J.M.B.: The EFNR is a very important society. As we said before, bridging basic and translational science with clinical science is essential. I believe it is highly important that the field of neurorehabilitation is examined and supported so extensively by a dedicated organization. However, it is equally crucial that these ideas, perspectives, concepts, and future directions are brought back to the larger community—here, I’m thinking of organizations such as the World Federation of Neurology, and specifically in Europe, the European Academy of Neurology. It is very important that this knowledge is shared with these bodies because neurorehabilitation is not only for specialists in neurorehabilitation; neurologists in general should also understand its importance and be able to guide their patients and teams in applying appropriate neurorehabilitative methods.

**S.D.: Based on your experience, how can we improve the rehabilitation of patients with Huntington's disease and what are the most common mistakes to be avoided?**

J.M.B.: That is a very interesting question. Indeed, what I said before in general terms is also valid for Huntington's disease. In Huntington's, there is a huge complexity in its phenomenology over the course of the disease. It can vary widely—starting with juvenile Huntington's in children, all the way up to cases in people around age 70 when the disease first manifests. It's the same genetic mutation, but the presentation is completely different. Therefore, it is crucial to target and personalize the different aspects of rehabilitation.

The second problem is the shortage of clinicians experienced in Huntington's disease. Professionals familiar with Parkinson's disease or stroke may not fully grasp the issues and challenges that people with Huntington's face. And this leads to my third point: it is highly important in Huntington's disease to have very strong empathy. Knowledge about the disease is essential. For example, it happens quite frequently that a patient says, “I have Huntington's disease,” and the doctor, physiotherapist, or speech therapist responds, “What is Huntington's disease?” So, the patient or family ends up having to explain it to them. Very often, the care provider reacts with shock—"What is Huntington's disease? This is a terrible disease." If the patient senses this shock, they can become completely blocked, and no progress can be made.


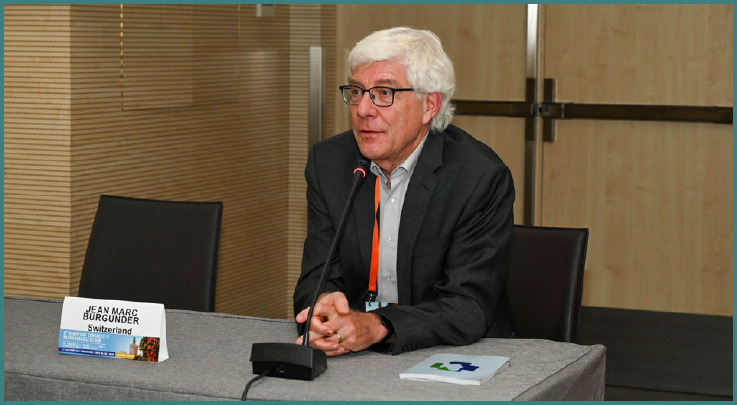



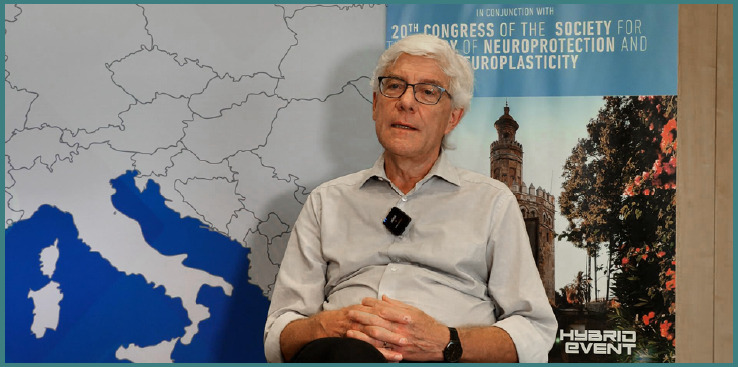


So, improving knowledge about the disease and increasing empathy among those who care for patients with Huntington's disease and their families is, from my perspective, a very big challenge.

